# Revisiting beta‐2 microglobulin as a prognostic marker in diffuse large B‐cell lymphoma

**DOI:** 10.1002/cam4.7239

**Published:** 2024-06-18

**Authors:** Jelena Jelicic, Karen Juul‐Jensen, Zoran Bukumiric, Mikkel Runason Simonsen, Michael Roost Clausen, Ahmed Ludvigsen Al‐Mashhadi, Robert Schou Pedersen, Christian Bjørn Poulsen, Anne Ortved Gang, Peter Brown, Tarec Christoffer El‐Galaly, Thomas Stauffer Larsen

**Affiliations:** ^1^ Department of Hematology Odense University Hospital Odense Denmark; ^2^ Faculty of Medicine Institute for Medical Statistics and Informatics, University of Belgrade Belgrade Serbia; ^3^ Department of Hematology, Clinical Cancer Research Center Aalborg University Hospital Aalborg Denmark; ^4^ Department of Hematology Vejle Hospital, Sygehus Lillebaelt Vejle Denmark; ^5^ Department of Hematology Aarhus University Hospital Aarhus Denmark; ^6^ Department of Hematology Regional Hospital Gødstrup Herning Denmark; ^7^ Department of Hematology Zealand University Hospital Roskilde Denmark; ^8^ Department of Clinical Medicine University of Copenhagen Kobenhavn Denmark; ^9^ Department of Hematology Copenhagen University Hospital, Rigshospitalet Copenhagen Denmark; ^10^ Department of Clinical Research University of Southern Denmark Odense Denmark

**Keywords:** non‐Hodgkin lymphoma, prognosis, prognostic factors, risk model

## Abstract

**Background:**

Several clinical prognostic models for diffuse large B‐cell lymphoma (DLBCL) have been proposed, including the most commonly used International Prognostic Index (IPI), the National Comprehensive Cancer Network IPI (NCCN‐IPI), and models incorporating beta‐2 microglobulin (β2M). However, the role of β2M in DLBCL patients is not fully understood.

**Methods:**

We identified 6075 patients with newly diagnosed DLBCL treated with immunochemotherapy registered in the Danish Lymphoma Registry.

**Results:**

A total of 3232 patients had data available to calculate risk scores from each of the nine considered risk models for DLBCL, including a model developed from our population. Three of four models with β2M and NCCN‐IPI performed better than the International Prognostic Indexes (IPI, age‐adjusted IPI, and revised IPI). Five‐year overall survival for high‐ and low‐risk patients were 43.6% and 86.4% for IPI and 34.9% and 96.2% for NCCN‐IPI. In univariate analysis, higher levels of β2M were associated with inferior survival, higher tumor burden (advanced clinical stage and bulky disease), previous malignancy and increased age, and creatinine levels. Furthermore, we developed a model (β2M‐NCCN‐IPI) by adding β2M to NCCN‐IPI (c‐index 0.708) with improved discriminatory ability compared to NCCN‐IPI (c‐index 0.698, *p* < 0.05) and 5‐year OS of 33.1%, 56.2%, 82.4%, and 96.4% in the high, high‐intermediate, low‐intermediate and low‐risk group, respectively.

**Conclusion:**

International Prognostic Indices, except for NCCN‐IPI, fail to accurately discriminate risk groups in the rituximab era. β2M, a readily available marker, could improve the discriminatory performance of NCCN‐IPI and should be re‐evaluated in the development setting of future models for DLBCL.

## Graphical abstract

Beta‐2 microglobulin (β2M) has been sporadically incorporated in prognostic models for diffuse large B‐cell lymphoma (DLBCL). We compared the prognostic discriminative ability of four existing DLBCL models with International Prognostic Indices. Moreover, we developed and validated a new model from the largest population investigating this marker so far, indicating the necessity to include β2M in future studies when analyzing prognostic markers in DLBCL.

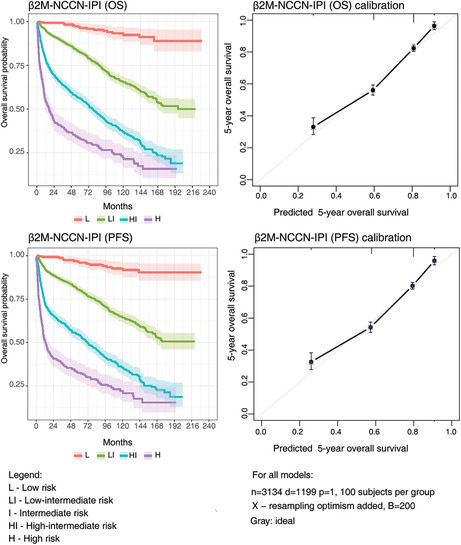

## 1 INTRODUCTION

Diffuse large B‐cell lymphoma (DLBCL) is the most frequent type of non‐Hodgkin lymphoma, accounting for approximately 30% of all lymphoid malignancies.^1^ DLBCL is characterized by significant heterogeneity in survival, and 30%–40% of patients are primary refractory or relapse following standard therapy.^2^ Accurate estimation of outcomes after standard therapy is of interest to tailor therapies and experimental approaches to patients' risk profiles.

The International Prognostic Index (IPI) was developed in 1993 as a prognostic tool for patients with aggressive lymphoma treated with doxorubicin.^3^ Patients were stratified into four risk groups based on five simple variables: age, Ann Arbor stage, Eastern Oncology Cooperative Group performance status (ECOG PS), number of extranodal sites, and lactate dehydrogenase (LDH). With the introduction of rituximab, several studies indicated suboptimal predictive power of the IPI in rituximab‐treated patients.^4^ The National Comprehensive Cancer Network IPI (NCCN‐IPI), proposed in 2014, is one of the most prominent models developed for DLBCL patients in the rituximab era.^5^ This model is based on the same five variables as the IPI but with four strata for age and three for LDH levels and refinement of high‐risk localizations. The prognostic superiority of NCCN‐IPI compared to other clinical models has been confirmed in several studies, further challenging the most widely adopted IPI as a prognostic tool in practice and clinical trials.^5^, ^6^, ^7^ However, NCCN‐IPI does not include markers that reflect the molecular‐genetic or other fingerprints of the tumor or the associated microenvironment, even though many new prognostic factors have been associated with survival, including clinical, radiologic, biological, cytogenetic, and molecular markers.^5^ However, including more sophisticated biomarkers in models for routine daily practice is difficult as they are time‐consuming to measure and associated with added costs.^8^

Many readily available biomarkers have been associated with survival in lymphoma patients, including beta‐2 microglobulin (β2M), a small polypeptide light chain that forms part of the major histocompatibility complex (MHC) class I antigens.^8^ The β2M is encoded by the *B2M* gene, which can be altered by different mechanisms, leading to impaired β2M protein expression. Additionally, *B2M* gene alterations can accumulate during cancer progression, contributing to poor reaction to cancer immunotherapies by dampening antigen presentation. In lymphoid malignancies, elevated serum β2M levels have been found in patients with significant tumor burden because β2M is ubiquitously expressed in most nucleated tumor cells, and white‐blood cell membrane turnover is the primary source of serum β2M.^8^ β2M has been related to inferior survival in patients with multiple myeloma and is part of the International Scoring System (ISS) and Revised ISS (R‐ISS).^9^, ^10^ In lymphoma patients, β2M is included in the Follicular Lymphoma International Prognostic Index 2 (FLIPI2) and PRIMA‐Prognostic Index (PRIMA‐PI).^11^, ^12^, ^13^ The prognostic significance of β2M has also been investigated in patients with DLBCL treated with and without rituximab.^14^, ^15^ Already in 2000, Conconi et al. tried to improve the prognostic value of the IPI and developed a prognostic model combining β2M and IPI variables (β2M‐IPI) in DLBCL patients treated with CHOP (cyclophosphamide, doxorubicin, vincristine, and prednisone)‐based regimes.^15^ Over the years, several models with β2M were primarily developed from limited populations.^16^, ^17^, ^18^ The most extensive study to date used to develop and validate a model with β2M came from a Spanish group (Grupo Español de Linfomas y Trasplantes de Médula Ósea—GELTAMO) that developed GELTAMO‐IPI in a population of 1848 patients treated with rituximab‐based regimes.^8^, ^19^ Attempts to combine β2M with clinical, laboratory immunohistochemical, and molecular markers have been made, but the validation of these models is lacking.^8^, ^20^, ^21^

We conducted a population registry‐based study to evaluate the prognostic role of β2M in, so far, the largest number of DLBCL patients. We aimed to (1) investigate the association of β2M with other clinically relevant prognostic factors in DLBCL registered in our national database, (2) compare four β2M‐based models with four International Prognostic Indices (IPI, age‐adjusted IPI [aaIPI], revised IPI [R‐IPI], NCCN‐IPI), and (3) investigate whether the addition of β2M to NCCN‐IPI would improve the discriminatory performance of NCCN‐IPI by developing a new model based on NCCN‐IPI variables and β2M.

## 2 METHODS

### 2.1 Patients

Patients analyzed in this study were identified through the nationwide Danish lymphoma registry (LYFO) as part of the Danish validation project.^22^ Patients newly diagnosed with DLBCL aged 18 years and older, diagnosed between January 2000 and June 2021, and treated with at least one cycle of rituximab plus CHOP (R‐CHOP)/R‐CHOP‐like therapy were screened for inclusion. Only patients with all clinical and laboratory variables required by analyzed prognostic models were included in the final analysis. Patients with testicular involvement, systemic disease, and concomitant CNS involvement were included in the study. Patients were excluded if they were diagnosed with primary central nervous system (CNS) lymphoma.

Prognostic models of interest were identified through a literature search and a previous systematic review.^4^ Eight models, including four International Prognostic Indices (IPI, aaIPI, R‐IPI, and NCCN‐IPI) and four models incorporating β2M were calculated using individual variables as provided in original publications.^3^, ^5^, ^15^, ^16^, ^17^, ^19^, ^23^

### 2.2 Statistical analysis

The primary survival outcome was OS measured from diagnosis until death from any cause or censoring at the last follow‐up. Progression‐free survival (PFS) was calculated from diagnosis until relapse/disease progression, death, or censoring at the last follow‐up. Survival was estimated using the Kaplan–Meier estimator, and differences between survival curves were tested using the log‐rank method. Cox proportional hazard models estimated associations between individual variables and OS and PFS. Logistic regression was used to measure the effects of explanatory variables on β2M, which was dichotomized according to the upper limit of normal (≤ULN vs. >ULN).

Integrated Brier score (IBS) was used to measure overall performance, with 0 indicating that prediction and outcome are equal and 1 indicating discordant prediction.^24^ Akaike information criterion (AIC) and Bayesian information criterion (BIC) were used as measures of fitness, with lower values indicating better fit.^25^ Moreover, the area under the receiver operating characteristic curve (AUC) was used to assess the discrimination of the predictive models.^26^ Discrimination (the model's ability to distinguish individuals with and without outcomes of interest) was also evaluated using the concordance index (0.5 indicates no discrimination, 1 indicates perfect discrimination) and Concordance Probability Estimate (higher values indicate better discrimination).^27^, ^28^, ^29^ Calibration (agreement between predicted and actual probabilities estimated by a predictive model) was presented with calibration curves. Models close to a 45‐degree line show perfect calibration.^30^

Interrater‐weighted *κ* statistics and 95% confidence intervals (CI) were used to compare agreement between the NCCN‐IPI and other four‐risk models.^31^ We used *t*‐test, Mann–Whitney, or chi‐square tests when appropriate to test the hypothesis for differences.

To develop a new model incorporating β2M, the population was randomly split into a training cohort comprising two‐thirds of the analyzed population and a validation cohort using the other one‐third of the population. The cut‐off for β2M, according to ULN, was chosen as previously reported by the GELTAMO group that developed the model with β2M from the largest patient population published so far. NCCN‐IPI variables were combined with β2M (≤ULN vs. >ULN), resulting in the risk score (β2M‐NCCN‐IPI) ranging from 0 to 9 points. Patients were regrouped into low (0–1 points), low‐intermediate (2–4), high‐intermediate (5–6), and high (>6 points) risk groups (Table S1). Several regrouping combinations in the training cohort were tested, with the one producing the highest c‐index selected. Only patients with all available variables of interest required by analyzed models were included, as multiple imputations would not reasonably approximate the true distributional relation between unobserved data and available information.^32^

All *p*‐values were two‐sided, and *p* < 0.05 was considered statistically significant. All analyses were performed in IBM SPSS Statistics 22 (IBM Corporation, Armonk, NY, USA), including randomization when developing the model and R‐4.0.0 software (The R Foundation for Statistical Computing, Vienna, Austria) using the following packages: CPE, ggplot2, ggsurvfit, dynpred, maxstat, pec, rms, survC1, and survival.

## 3 RESULTS

**FIGURE 1:**
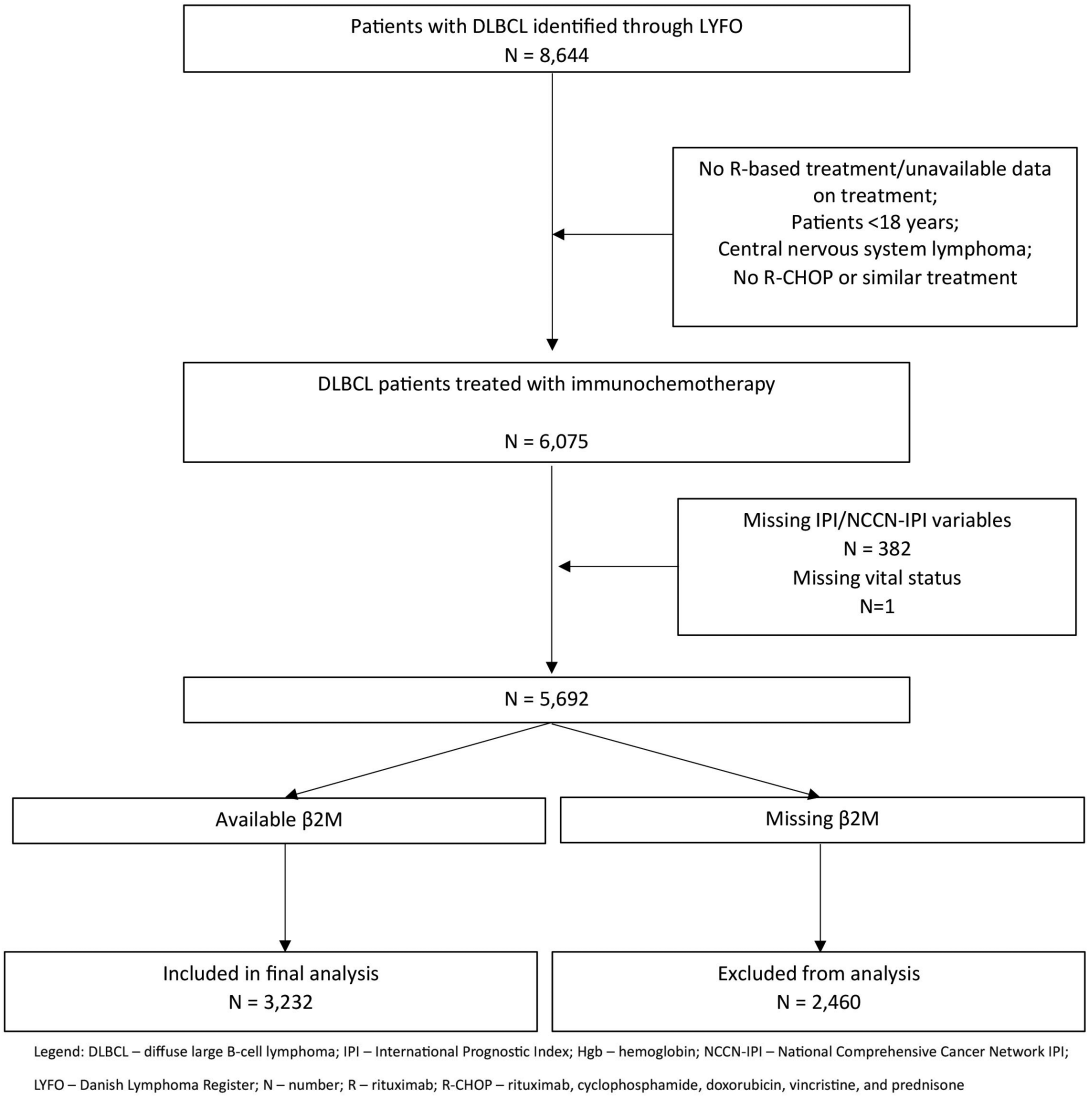
Consort diagram of the selection process for identifying patients eligible for the current study.

Of 6075 patients with DLBCL registered in LYFO who received at least one cycle of R‐CHOP/R‐CHOP‐like therapy in the inclusion period, only 382 patients lacked IPI/NCCN‐IPI variables, while one lacked data on vital status. Among 5692 potential candidates, data to calculate required prognostic indices of interest were available for 3232, while 2460 patients were excluded due to missing data on β2M (Figure 1).

The median age of the analyzed population was 68 years (range 18–95), with 71.3% older than 60. Table 1 summarizes the baseline patient characteristics of 3232 patients included in the final analysis. Additionally, clinical characteristics of the excluded patients with available IPI/NCCN‐IPI parameters are provided as comparisons. Of note, among 2460 excluded patients (43.2%), there was no difference regarding age and gender compared to patients included in the final analysis. However, differences were observed regarding other analyzed variables, as provided in Table 1.

**TABLE 1:** Clinical characteristics of patients with diffuse large B‐cell lymphoma.

Patient characteristics	N of patients included in the main analysis *N* = 3232	N of patients excluded due to missing β2M *N* = 2460	*p*‐value
Age			
Median (range)	68 (18–95)	68 (18–95)	0.979
≤40	178 (5.5%)	138 (5.6%)	0.836
41–60	750 (23.2%)	579 (23.5%)	
61–75	1493 (46.2%)	1104 (44.9%)	
>75	811 (25.1%)	639 (26.0%)	
Gender			
Males	1819 (56.3%)	1416 (57.6%)	0.334
Ann Arbor stage			
I	559 (17.3%)	380 (15.4%)	0.001
II	518 (16.0%)	339 (13.8%)	
III	582 (18.0%)	446 (18.1%)	
IV	1573 (48.7%)	1295 (52.6%)	
ECOG PS			
≥2	480 (14.9%)	511 (20.8%)	<0.001
LDH			
≤ULN	1536 (47.5%)	998 (40.6%)	<0.001
>1 to ≤3xULN	1434 (44.4%)	1154 (46.9%)	
>3xULN	242 (8.1%)	308 (12.5%)	
EN IPI			
>1	987 (30.5%)	790 (32.1%)	0.204
EN NCCN‐IPI			
≥1	991 (30.7%)	872 (35.4%)	<0.001
β2M			
>ULN	1746 (54.0%)	ND	ND
Bulky disease			
(≥10 cm)	974 (30.1%)	782 (31.8%)	0.012
ND	88 (2.7%)	175 (7.1%)	
Creatinine, μmol/L			
Median (range)	75 (19–950)	77 (23–753)	0.394
ND	177 (5.5%)	263 (10.7%)	
Other malignancy^a^			
Yes	361 (11.2%)	306 (12.4%)	0.023
ND	184 (5.7%)	269 (10.9%)	
Therapy^b^			
R‐CHOP	2755 (85.2%)	2047 (83.2%)	0.037
R‐CHOEP	313 (9.7%)	244 (9.9%)	
R‐CEOP	62 (1.9%)	57 (2.3%)	
DA EPOCH‐R	35 (1.1%)	42 (1.7%)	
R‐miniCHOP	12 (0.4%)	14 (0.6%)	
Other	55 (1.7%)	56 (2.3%)	

Abbreviations: ECOG PS, Eastern Oncology Cooperative Group performance status; EN, extranodal; IPI, International Prognostic Index; LDH, lactate dehydrogenase; N, number; NCCN‐IPI, National Comprehensive Cancer Network‐IPI; ND, no data; ULN, upper limit of normal; β2M, beta‐2 microglobulin.

^a^ Excluding skin cancer other than melanoma.

^b^ Treatment regimes: R‐CHOP—rituximab, cyclophosphamide, doxorubicin, vincristine, prednisone; R‐CHOEP—rituximab, cyclophosphamide, doxorubicin, vincristine, etoposide, prednisone; R‐CEOP—rituximab, cyclophosphamide, vincristine, etoposide, prednisone; dose‐adjusted EPOCH‐R—rituximab, cyclophosphamide, doxorubicin, vincristine, etoposide, prednisone; R‐miniCHOP—rituximab, cyclophosphamide (dose‐reduced), doxorubicin (dose‐reduced), vincristine (dose‐reduced), prednisone.

Among 5692 potential candidates, a higher percentage of patients diagnosed between 2000 and 2010 lacked reports on β2M (45.0% with vs. 55.0% without data on β2M) compared to those diagnosed from 2011 to 2021 (38.8% without vs. 61.2% with data on β2M, *p* < 0.001).

### 3.1 Prognostic models

Table S1 summarizes the variables included in each model along with distributions of patients according to risk categories in original models for a more straightforward overview. Table 2 provides distributions of patients from our cohort concerning risk groups and 3‐ and 5‐year PFS and OS according to each model.

**TABLE 2:** Distribution of patients within risk groups of analyzed prognostic models for Diffuse large B‐cell lymphoma patients concerning 3‐ and 5‐year overall and progression‐free survival.

Model (N of patients)	Risk groups	N of patients (%)	3‐year PFS (%)	5‐year PFS (%)	3‐year OS (%)	5‐year OS (%)
IPI^3^	L	902 (27.9)	91.0	84.7	92.7	86.4
	LI	813 (25.2)	79.8	73.9	81.9	75.6
	HI	873 (27.0)	69.5	63.4	72.9	65.8
	H	644 (19.9)	46.8	43.0	48.9	43.6
aaIPI^3^	L	698 (21.6)	88.2	80.8	89.9	82.5
	LI	815 (25.2)	79.6	72.7	82.3	74.8
	HI	1134 (35.1)	68.5	64.2	71.4	66.3
	H	585 (18.1)	57.5	53.4	59.1	53.5
R‐IPI^23^	Very good	232 (7.2)	98.6	96.1	99.1	96.6
	Good	1483 (45.9)	83.6	76.9	85.8	78.8
	Poor	1517 (46.9)	59.7	54.7	62.7	56.4
NCCN‐IPI^5^	L	274 (8.5)	98.5	95.7	98.9	96.2
	LI	1245 (38.5)	86.4	80.8	89.0	83.1
	HI	1310 (40.5)	66.7	59.9	69.3	61.7
	H	403 (12.5)	39.2	34.5	41.2	34.9
β2‐IPI^15^	L	685 (21.2)	94.0	89.1	95.5	90.7
	LI	617 (19.1)	85.4	79.2	87.9	80.9
	HI	734 (22.7)	76.6	69.8	78.6	71.8
	H	1196 (37.0)	53.8	48.4	56.8	50.8
GELTAMO‐IPI^19^	L	286 (8.8)	97.9	94.9	98.3	95.4
	LI	1972 (61.0)	82.1	76.3	84.6	78.4
	HI	574 (17.8)	59.4	52.0	63.0	53.8
	H	400 (12.4)	34.2	28.9	35.3	29.5
Modified Prognostic Model^17^	L	228 (7.1)	98.6	95.4	98.3	96.0
	LI	551 (17.0)	92.9	88.1	84.6	89.6
	HI	1529 (47.3)	76.6	69.8	63.0	72.0
	H	924 (28.6)	50.6	45.6	35.3	47.0
New Prognostic Index^16^	L	359 (11.1)	97.2	94.7	97.7	95.3
	LI	1882 (58.2)	81.9	75.8	84.3	77.6
	HI	728 (22.5)	53.7	47.3	57.1	50.0
	H	263 (8.1)	35.4	29.7	37.6	30.0
β2M‐NCCN‐IPI	L	258 (8.0)	98.8	95.9	99.2	96.4
	LI	1556 (48.1)	85.9	80.3	88.6	82.4
	HI	1057 (32.7)	61.3	54.4	63.9	56.2
	H	361 (11.2)	37.9	32.6	39.7	33.1

Abbreviations: aaIPI, age‐adjusted IPI; GELTAMO‐IPI, Grupo Español de Linfomas y Trasplantes de Médula Ósea IPI; H, high; HI, high‐intermediate; IPI, International Prognostic Index; L, low; LI, low‐intermediate; NCCN‐IPI, National Comprehensive Cancer Network‐IPI; N, number; OS, overall survival; PFS, progression‐free survival; R‐IPI, Revised International Prognostic Index; ULN, upper limit of normal; β2M, beta‐2 microglobulin.

### 3.2 Variables included in models and correlations with β2M

The disease stage was the only IPI parameter used in all models, while all models except aaIPI included age in the model. Moreover, gradations into several age groups were used in NCCN‐IPI and GELTAMO‐IPI. ECOG PS and LDH were used in seven models, with the gradation of LDH used only in NCCN‐IPI. Extranodal localizations were combined with other parameters in five models.

β2M was part of four previously reported models, with two models dichotomizing β2M according to the ULN and two according to an optimal cutoff (Table S1).

Among all analyzed parameters (age, stage, ECOG PS, extranodal localizations, LDH, previous malignancy, bulky disease, and creatinine levels), only gender was not associated with β2M levels. When excluding gender from further analysis, multivariate logistic regression analysis showed that all parameters influenced levels of β2M (Table S2).

### 3.3 Model agreement

Regarding our cohort of interest, IPI classified 27.9% of patients into low‐risk groups, whereas 19.9% were in high‐risk group. R‐IPI, NCCN‐IPI, and GELTAMO‐IPI classified 7.2%, 8.5%, and 8.8%, respectively, in the low‐risk group and 46.9%, 12.5%, and 12.4% in the high‐risk group.

As presented in Table S3, we analyzed the agreement between the NCCN‐IPI as the reference model and other models stratifying patients into four risk groups. Based on the weighted κ analysis, substantial agreement (weighted *κ* between 0.61 and 0.80) was only observed between NCCN‐IPI and the IPI (0.630) and GELTAMO‐IPI (0.612).

### 3.4 Survival

#### 3.4.1 Overall survival (OS)

The median follow‐up of the study population was 59.5 months (range 0.30–228.6 months). There were 1283 deaths (39.4%). The median survival of the whole study population was 142.8 months (95% CI 131.5–154.0).

Parameters included in each model were prognostically significant in univariate analysis (Table 3). However, when β2M was combined with individual IPI variables, EN localizations lost prognostic significance. On the contrary, multivariate analysis with β2M and NCCN‐IPI variables showed that all parameters retained prognostic significance.

**TABLE 3:** Univariate and multivariate analysis of IPI/NCCN‐IPI variables and β2M concerning overall survival.

Variables	Univariate analysis		Multivariate analysis with IPI variables and β2M		Multivariate analysis with NCCN‐IPI variables and β2M	
	HR, 95 %CI	*p*‐value	HR, 95% CI	*p*‐value	HR, 95% CI	*p*‐value
Age ≤ 60 vs. >60 years	4.143 (3.506; 4.896)	<0.001	3.440 (2.902; 4.077)	<0.001		
Age ≤ 40	Reference				Reference	
41–60	4.261 (2.175; 8.348)	<0.001			3.539 (1.805; 6.938)	<0.001
61–75	11.322 (5.861; 21.870)	<0.001			8.728 (4.511; 16.885)	<0.001
>75	25.960 (13.415; 50.236)	<0.001			17.427 (8.972; 33.848)	<0.001
Stage I/II vs. III/IV	1.863 (1.641; 2.115)	<0.001	1.165 (1.009; 1.344)	0.037	1.195 (1.035; 1.380)	0.015
ECOG PS 0–1 vs. ≥2	3.087 (2.723; 3.499)	<0.001	2. 211 (1.938; 2.522)	<0.001	1.975 (1.728; 2.257)	<0.001
N of EN sites 1 vs. >1	1.418 (1.264; 1.591)	<0.001	1.049 (0.928; 1.185)	0.448		
EN sites (NCCN‐IPI) <1 vs. ≥1	1.538 (1.372; 1.724)	<0.001			1.136 (1.005; 1.284)	0.041
LDH IPI normal vs. >ULN	1.497 (1.339; 1.673)	<0.001	1.141 (1.013; 1.285)	0.030		
LDH ≤ ULN	Reference				Reference	
>1 to ≤3xULN	1.234 (1.234; 1.558)	<0.001			1.226 (1.083; 1.388)	0.001
>3xULN	2.223 (1.852; 2.669)	<0.001			1.430 (1.177; 1.737)	<0.001
β2M > ULN yes vs. no	3.135 (2.776; 3.540)	<0.001	2.137 (1.875; 2.434)	<0.001	1.833 (1.605; 2093)	<0.001
β2M <3.2 mg/L yes vs. no	3.020 (2.702; 3.376)	<0.001				
β2M <2.5 mg/L yes vs. no	2.975 (2.638; 3.354)	<0.001				

Abbreviations: CI, confidence interval; ECOG PS, Eastern Oncology Cooperative Group performance status; EN, Extranodal; GELTAMO‐IPI, Grupo Español de Linfomas y Trasplantes de Médula Ósea IPI; HR, hazard ratio; IPI, International Prognostic Index; LDH, lactate dehydrogenase; NCCN‐IPI—National Comprehensive Cancer Network‐IPI; N, number; ULN, upper limit of normal; β2M, beta‐2 microglobulin.

**FIGURE 2:**
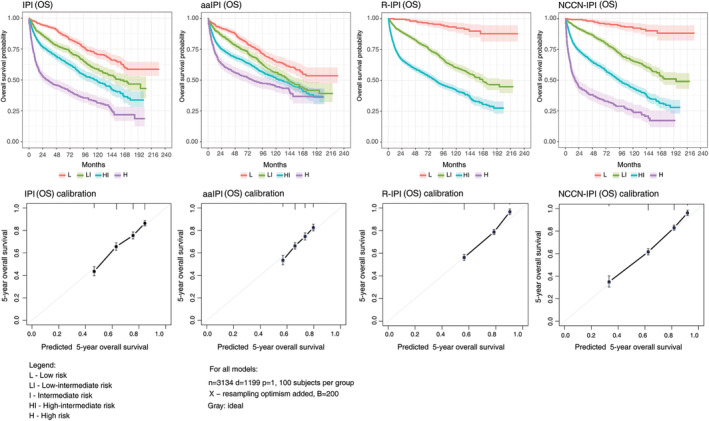
Kaplan–Meier and calibration curves of four International Prognostic Indices in diffuse large B‐cell lymphoma patients with respect to overall survival. The shaded color areas around curves represent confidence intervals.

**FIGURE 3:**
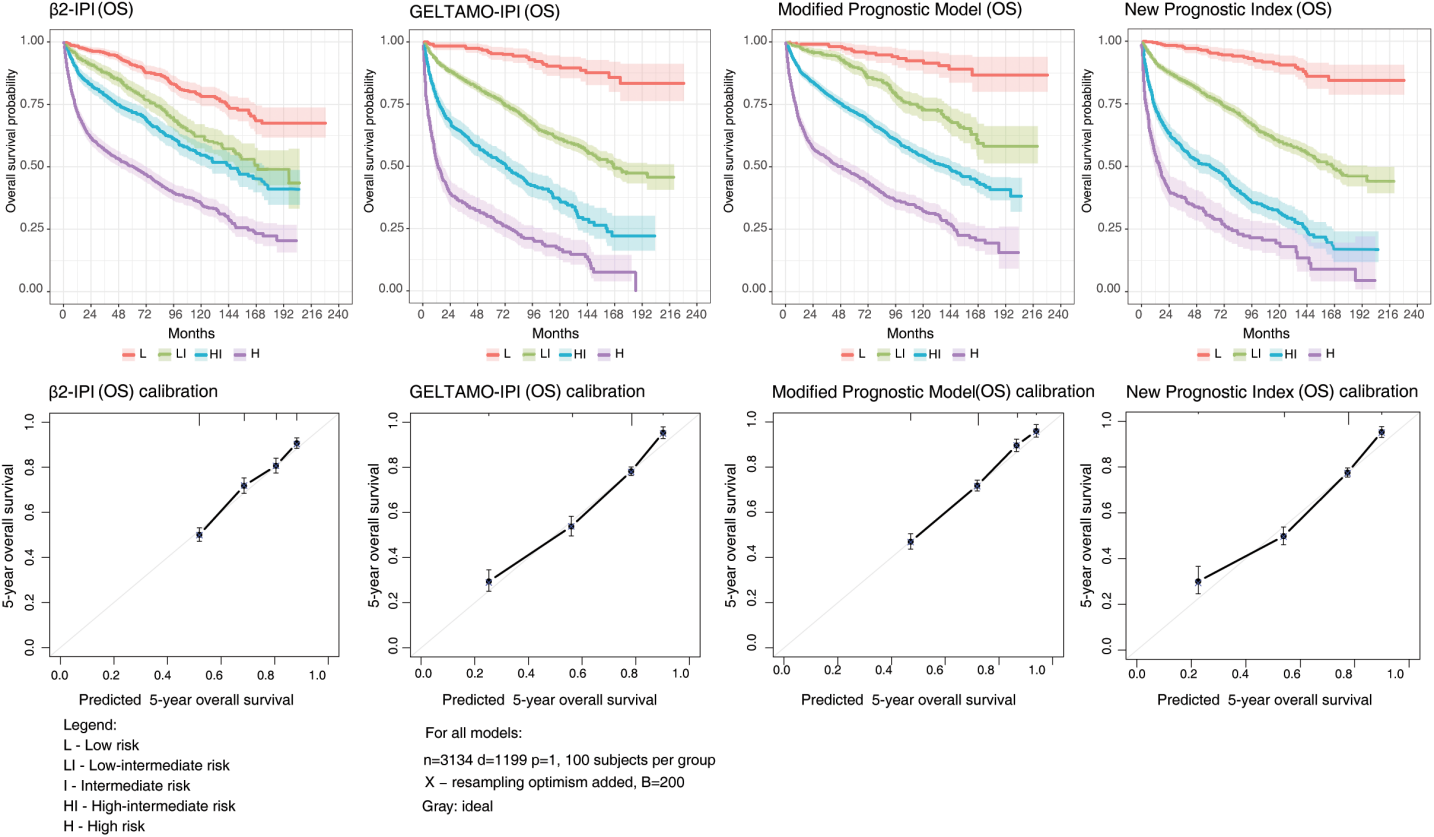
Kaplan–Meier and calibration curves of four models with β2M in diffuse large B‐cell lymphoma patients with respect to progression‐free survival. The shaded color areas around curves represent confidence intervals.

Figure 2 presents Kaplan–Meier and calibration curves for International Prognostic Indices, while Figure 3 presents the same for models with β2M. Table 3 summarizes calculated 3‐ and 5‐year OS rates for all models. Although most models did not identify patients with a high risk of poor survival, only GELTAMO‐IPI could provide 5‐year OS estimates below 30%. NCCN‐IPI came very close with a 5‐year OS estimate of 34.9%, significantly lower than the survival percentages obtained for high‐risk IPI patients (43.6%) and R‐IPI patients (56.4%). In the respective low‐risk groups, 5‐year OS was 95.4% and 96.2% for GELTAMO‐IPI and NCCN‐IPI, while a lower estimate of 86.4% was registered for IPI, but not for R‐IPI (96.6%) (Table 2).

#### 3.4.2 Progression‐free survival (PFS)

The median PFS was 139.8 months (95% CI, 128.3–151.3 months). Table S4 provides hazard ratios (HRs) for PFS for risk groups within each prognostic model. Kaplan–Meier and calibration curves were similar to those of OS (data not provided).

Regarding 2460 patients with missing data on β2M required to calculate models incorporating β2M, the median follow‐up was 59.5 months (0.07–266.8), with 1217 deaths (49.5%) during the follow‐up. Median PFS was 103.7 (95% CI 92.1–115.4), and OS was 112 months (95% CI 102.1–123.7). There was a statistical difference between patients without and with available β2M concerning PFS and OS (*p* < 0.001).

### 3.5 Model fit, discrimination, and calibration

As a measure of overall performance, we estimated the IBS, with the lowest value regarding OS calculated for GELTAMO‐IPI (0.183), followed by New Prognostic Index (0.184), and NCCN‐IPI (0.188) (Table 4). As measures of model fit, we calculated AIC and BIC with lower values, indicating better model quality. The lowest AIC was registered for GELTAMO‐IPI (18708), New Prognostic Index (18737), and NCCN‐IPI (18806). The highest AIC was registered for aaIPI (19257) (Table 4).

**TABLE 4:** Summary of hazard ratios, overall performance, fit/quality, and discrimination measures concerning overall survival.

	HR (95% CI)	IBS	AIC	BIC	CPE	AUC	c‐index	Difference in c‐index with NCCN‐IPI as referent model
IPI^3^	Reference	0.198	19,010	19,015	0.641	0.655	0.677 (0.665; 0.690)	−0.021 (−0.029; −0.012)
	1.650 (1.382; 1.970)							
	2.379 (2.012; 2.812)							
	4.649 (3.935; 5.493)							
aaIPI^3^	Reference	0.212	19,257	19,272	0.583	0.604	0.618 (0.597; 0.639)	−0.080 (−0.093; −0.067)
	1.418 (1.186; 1.696)							
	1.826 (1.549; 2.152)							
	2.542 (2.123; 3.042)							
R‐IPI^23^	Reference	0.198	19,028	19,038	0.629	0.630	0.646 (0.631; 0.661)	−0.052 (−0.060; −0.045)
	6.18 (3.755; 10.170)							
	13.02 (7.932; 21.370)							
NCCN‐IPI^5^	Reference	0.188	18,806	18,821	0.670	0.675	0.698 (0.686; 0.710)	Reference
	5.274 (3.284; 8.469)							
	11.606 (7.261; 18.550)							
	25.534 (15.832; 41.179)							
β2‐IPI^15^	1.956 (1.562; 2.448)	0.193	18,919	18,934	0.657	0.669	0.688 (0.677; 0.699)	−0.010 (−0.020; −0.001)
	2.647 (2.144; 3.268)							
	5.425 (4.481; 6.568)							
GELTAMO‐IPI^19^	Reference	0.183	18,708	18,723	0.660	0.677	0.697 (0.683; 0.711)	−0.001 (−0.011; 0.009)
	4.713 (3.183; 6.978)							
	11.003 (7.365; 16.436)							
	23.984 (16.042; 35.857)							
Modified Prognostic Model^17^	Reference	0.189	18,869	18,884	0.663	0.670	0.686 (0.670; 0.703)	−0.012 (−0.024; 0.001)
	3.405 (2.050; 5.654)							
	7.221 (4.457; 11.698)							
	16.299 (10.051; 26.430)							
New Prognostic Index^16^	Reference	0.184	18,737	18,752	0.668	0.676	0.693 (0.676; 0.711)	−0.005 (−0.020; 0.010)
	4.913 (3.425; 7.047)							
	13.041 (9.045; 18.803)							
	22.992 (15.717; 33.635)							
β2M‐NCCN‐IPI	Reference	0.185	18,744	18,479	0.662	0.669	0.708 (0.684; 0.733)	0.010 (0.005; 0.016)
	5.720 (3.474; 9.419)							
	15.212 (9.248; 25.021)							
	29.446 (17.744; 48.865)							

Abbreviations: aaIPI, age‐adjusted IPI; AIC, Akaike information criterion; AUC, area under the curve; BIC, Bayesian Information Criterion; CI, confidence interval; c‐index—concordance index; CPE, concordance probability estimate; DLBCL, diffuse large B‐cell lymphoma; GELTAMO‐IPI—Grupo Español de Linfomas y Trasplantes de Médula Ósea IPI; HR, hazard ratio; IBS, integrated Brier score; IPI, International Prognostic Index; NCCN‐IPI, National Comprehensive Cancer Network‐IPI; N—number; R‐IPI—Revised International Prognostic Index; β2M, beta‐2 microglobulin.

Of discrimination measures, the highest CPE values were found for NCCN‐IPI (0.670), New Prognostic Index (0.668), and Modified NCCN‐IPI (0.663). The lowest CPE was registered for aaIPI (0.583). Additionally, when AUC was calculated, GELTAMO‐IPI (0.677), New Prognostic Index (0.676), and NCCN‐IPI (0.675) showed the highest values, while the lowest AUC was found for aaIPI (0.603). Models that provided the highest c‐index were NCCN‐IPI (0.698), GELTAMO‐IPI (0.697), and New Prognostic Index (0.693). When these models were compared to NCCN‐IPI as the reference model, there was no statistical difference between the c‐indexes (*p* > 0.05). However, NCCN‐IPI discriminated significantly better than IPI (c‐index 0.677), aaIPI (0.618), and R‐IPI (0.646) (Table 4).

Similar results were obtained for PFS, in terms of overall performance measures, fitness, and discrimination, as for OS. Of note, all four models with β2M had higher c‐index than IPI, aaIPI, and R‐IPI but did not outperform NCCN‐IPI (Table S5).

### 3.6 Model development

**FIGURE 4:**
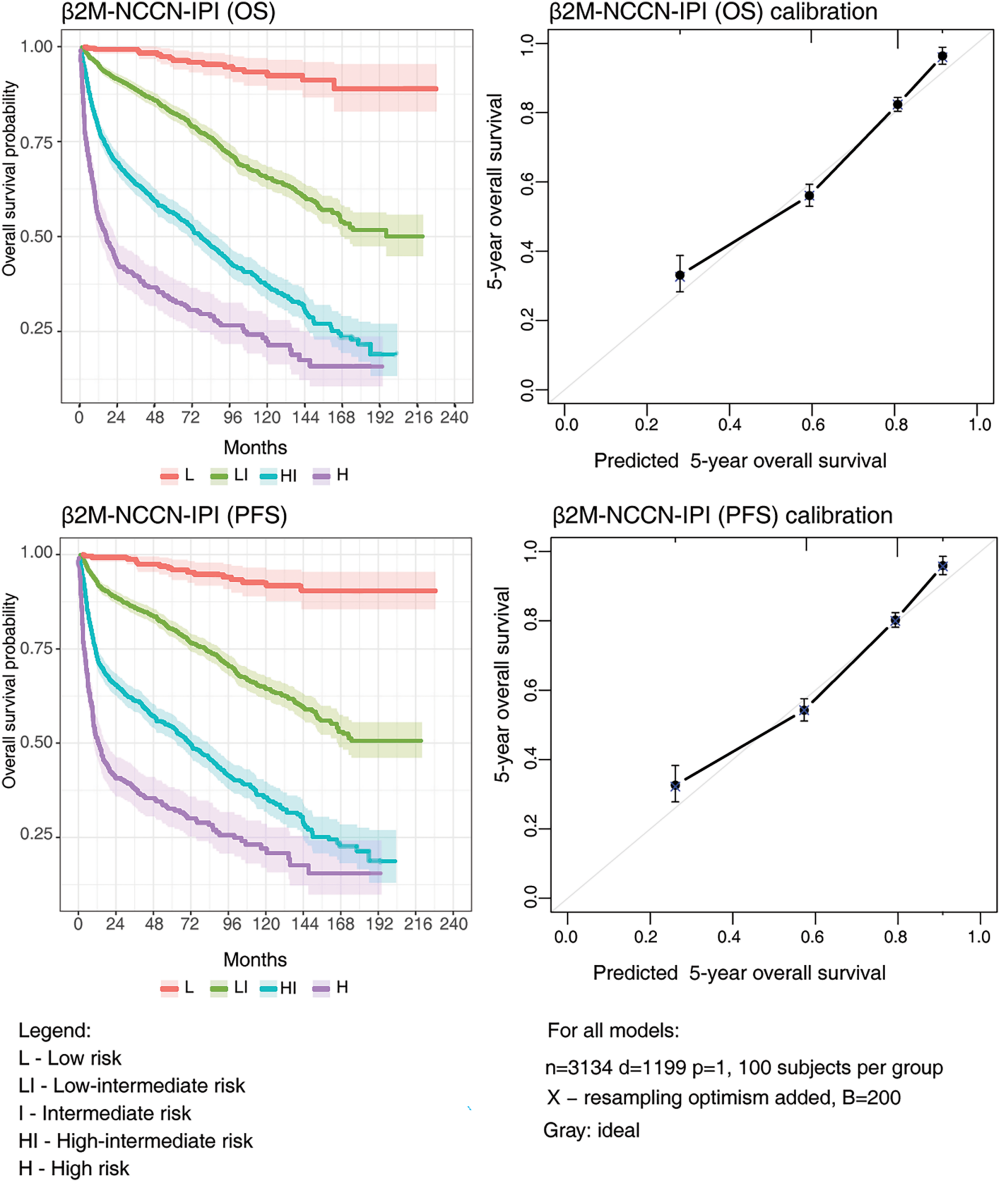
Kaplan–Meier and calibration curves of new β2M‐NCCN‐IPI with respect to overall and progression‐free survival. The shaded color areas around curves represent confidence intervals.

As provided in Table S6, the new model (β2M‐NCCN‐IPI) showed improved performance measures compared to NCCN‐IPI and IPI in both the training (2155 patients) and validation cohorts (1077 patients). When analyzing all 3232 patients together, they were categorized into low‐risk (8.0%), low‐intermediate (48.1%), high‐intermediate (32.7%), and high‐risk (11.2%) groups (Table 2). Although β2M‐NCCN‐IPI had almost perfect risk group agreement (weighted *κ* = 0.873) with NCCN‐IPI, this model provided a statistically significant improvement of the c‐index regarding PFS (0.700) and OS (0.708) compared to the NCCN‐IPI (Table 4; Table S4). The new model showed a more significant difference in survival rates between low‐ and high‐risk groups than NCCN‐IPI. The 5‐year overall survival was 33.1% in the high‐risk group and 96.4% in the low‐risk group. However, the new model could not accurately identify patients with very poor survival, but it provided improved estimates compared to NCCN‐IPI. (Table 2; Figure 4). Kaplan–Meier curves and calibration curves of the new model (β2M‐NCCN‐IPI) are presented in Figure 4.

## 4 DISCUSSION

In this extensive population‐based analysis of prognostic models, we confirmed the prognostic value of International Prognostic Indices (IPI, aaIPI, R‐IPI, and NCCN‐IPI) and four models that include β2M (β2‐IPI, GELTAMO‐IPI, Modified Prognostic Model, and New Prognostic Index). However, IPI, aaIPI, and R‐IPI showed statistically inferior performance measures compared to other models. Regarding PFS and OS, the discriminatory ability of NCCN‐IPI was improved compared to models with β2M, but it did not statistically outperform these models.^5^, ^19^, ^33^, ^34^ However, adding β2M to NCCN‐IPI improves the NCCN‐IPI's discriminatory ability.

One of the potentially readily available markers with prognostic significance in hematological malignancies is β2M, a simple and inexpensive laboratory biomarker that has shown prognostic significance in diverse lymphoproliferative disorders.^14^ Moreover, β2M can be increased in patients with systemic or local inflammation and those with renal failure, as it is mainly excreted by the kidneys.^8^ Measurement of β2M is part of the baseline work‐up in patients with multiple myeloma and follicular lymphoma, although the role of β2M as an adverse prognostic factor in lymphoproliferative diseases is not fully understood.^8^, ^9^, ^10^, ^11^ β2M is an important unit of MHC class I and is essential for properly functioning the MHC class I heavy chain, enhancing the ability to bind peptides.^35^ In cancer patients, *B2M* gene alterations have been related to MHC class I deficiency and loss of β2M protein expression, facilitating tumor cell escape from the host's immune control. This avoidance mechanism, tumor evasion, was recognized as one of the critical processes of tumor resistance to the cytolytic activity of T cells.^36^ Additionally, β2M deficiency has been connected to immune escape in melanoma and non‐small‐cell lung cancer patients and related to unfavorable prognosis.^36^ Genetic alterations associated with the inactivation of the *B2M* gene were reported in 29% of DLBCL cases.^37^

Several studies have previously demonstrated the prognostic significance of β2M in DLBCL patients treated with and without rituximab.^1^, ^8^, ^14^, ^15^, ^19^, ^38^ In the original study behind the IPI, β2M was shown to be an independent prognostic marker, but due to many patients with missing values, this parameter was not incorporated in the final model.^3^ In an attempt to investigate whether the addition of β2M increases the prognostic value of the IPI, Conconi et al. proposed β2‐IPI.^15^ Due to missing values, this model was developed in only 71 patients treated with CHOP therapy.^15^ Montalbán et al. added a β2M to the main variables of IPI and improved risk assessment in DLBCL in a study that proposed GELTAMO‐IPI.^19^ Although parameters included in the IPI were significant in our univariate analysis, only extranodal sites lost their prognostic significance when adding β2M in multivariate analysis. Interestingly, GELTAMO‐IPI used β2M instead of extranodal sites, with improved discriminatory ability in the development (1230 patients) and validation settings (618 patients).^19^ Moreover, GELTAMO‐IPI was validated in several independent retrospective series involving between 116 and 439 patients.^1^, ^39^, ^40^, ^41^ Compared to β2‐IPI and GELTAMO‐IPI, which dichotomized β2M concerning the ULN, the other two models incorporated dichotomized β2M value according to the optimal cutoff evaluated by AUC. Kanemasa et al. proposed a four‐level model based on age, stage, ECOG PS, and β2M cutoff of 3.2 mg/L, while Kang et al. combined the same parameters with the addition of LDH and different β2M cutoff of 2.5 mg/L.^16^, ^17^ Both models were developed from retrospective studies with a limited number of patients (274 and 621, respectively).^16^, ^17^

Although all four‐level models could stratify patients into four risk groups, only three models could identify populations with poor outcomes and 5‐year survival of 35% or less in high‐risk groups, including GELTAMO‐IPI (29.5%), New Prognostic Model (30.0%), and NCCN‐IPI (34.9%). In comparison, the 5‐year OS for IPI was 42.6%. Regarding patients in the low‐risk group, NCCN‐IPI could identify most patients with excellent prognoses with a 5‐year OS of 96.2%, followed by Modified Prognostic Model (96.0%), GELTAMO‐IPI (95.4%), and New Prognostic Model (95.3%). R‐IPI showed good stratification ability of low‐risk patients with 5‐year survival comparable to NCCN‐IPI.

One of the most extensive validation studies involving 2124 patients from seven clinical trials revealed that all International Prognostic Indices had lower prognostic ability than initially reported.^42^ However, in our recent study comparing 13 models developed for DLBCL, including models combining IPI variables with different laboratory parameters, NCCN‐IPI consistently showed superior performance than other analyzed models.^7^ The study included 5126 patients with DLBCL, resulting in similar 5‐year survival rates to the original NCCN‐IPI. This may be due to both cohorts being representative of real‐life populations.^7^ As β2M frequently lacked, comparisons regarding model performance with models including β2M were not conducted. In the current analysis comparing performance measures among International Prognostic Indices and models incorporating β2M, we found superior discriminatory ability evaluated by the c‐index of NCCN‐IPI (0.698), GELTAMO‐IPI (0.697), and New Prognostic Index (0.693) compared to IPI (0.677), aaIPI (0.18), and R‐IPI (0.646). However, NCCN‐IPI did not statistically outperform models with β2M, nor did GELTAMO‐IPI show improved discriminatory ability compared to NCCN‐IPI. When other measures of model performance were calculated, including calibration concerning PFS and OS, NCCN‐IPI and β2M‐based models again provided better performance than other International Prognostic Indices.

Coutinho et al. conducted a validation study on 386 patients uniformly treated with R‐CHOP, concluding that NCCN‐IPI was better than IPI and GELTAMO‐IPI in identifying patients with a poor prognosis.^40^ The study found that when controlling for NCCN‐IPI risk groups, bulky disease and elevated β2M were independent predictors of poor prognosis. However, adding them to NCCN‐IPI did not improve the identification of patients with poor outcomes.^40^ To analyze whether β2M improves the prognostic significance of NCCN‐IPI in our cohort, we tested a model by adding β2M to NCCN‐IPI and regrouping patients into four risk groups. The new model named β2M‐NCCN‐IPI provided superior performance measures than other models with 5‐year survival in high‐ and low‐risk groups of 33.1% and 96.4%, respectively. The improvement of NCCN‐IPI by adding β2M reflects better regrouping, particularly in low‐intermediate and high‐intermediate groups, with a 5‐year survival of 56.2% and 82.4% in high‐intermediate and low‐intermediate groups, respectively, compared to 61.7% and 83.1% in the respective NCCN‐IPI groups. Moreover, β2M could add prognostic power to NCCN‐IPI due to the association of β2M to prognostically adverse markers such as increased age, advanced stage, extranodal involvement, increased LDH, bulky disease, secondary malignancy, and impaired kidney function. We confirm previous findings that β2M may reflect tumor burden, although other conditions could be contributing factors and should be cautiously evaluated.^21^

The current study's main limitation is the retrospective nature, and many excluded patients due to missing data on β2M, which could impact the results. Excluded patients were more often diagnosed in earlier years of the study selection period. Moreover, they tended to have more adverse IPI/NCCN‐IPI factors, although no differences regarding age and gender were observed. This finding may reflect a failure to complete all diagnostic and prognostic assessments, including β2M in patients presenting with high tumor volume, rapidly progressing disease, and negatively affected performance status in urgent need of therapy. As we lacked data on cell of origin and chromosomal translocations (MYC and BCL2 or/and BCL6), we were unable to compare models integrating biologic prognostic markers reflecting recent advances in DLBCL's genomics, molecular biology, immunology, and radiology, further limiting comparisons with other potentially relevant models.^8^, ^20^, ^21^ One limitation of the used registry is the lack of precise data on other malignancies, which hinders the thorough investigation of their effects on β2M elevation. However, the study aimed to compare four models integrating β2M with International Prognostic Indices, validate these models in the most extensive population analyzing β2M in DLBCL, and finally develop a model showing that β2M could further increase the discriminatory ability of existing models. Moreover, we compared patients with and without available β2M. By this, we confirm a potential selection bias when many patients are excluded for different reasons despite a large, selected population.

## 5 CONCLUSIONS

In this large retrospective nationwide register‐based study, we found superior model quality and discriminatory ability of NCCN‐IPI and four models incorporating β2M compared to IPI, aaIPI, and R‐IPI. Four models incorporating β2M did not outperform NCCN‐IPI in terms of performance measures. However, adding β2M to NCCN‐IPI could provide a superior discrimination ability of the newly proposed model (β2M‐NCCN‐IPI) than other models. Therefore, we suggest that β2M should be considered in future studies aiming to develop models for DLBCL as it is related to factors of prognostic importance for survival in lymphoma patients reflecting greater tumor burden (advanced clinical stage, extranodal sites, bulky disease, and serum LDH level) in addition to increased age and creatinine levels.

## AUTHOR CONTRIBUTIONS

**Jelena Jelicic:** Conceptualization (equal); data curation (equal); formal analysis (equal); methodology (equal); writing – original draft (lead); writing – review and editing (equal). **Karen Juul‐Jensen:** Conceptualization (equal); data curation (equal); methodology (equal); resources (equal); supervision (equal); writing – review and editing (equal). **Zoran Bukumiric:** Conceptualization (equal); formal analysis (equal); methodology (equal); supervision (equal); writing – review and editing (equal). **Mikkel Runason Simonsen:** Formal analysis (equal); methodology (equal); supervision (supporting); writing – review and editing (equal). **Michael Roost Clausen:** Conceptualization (equal); methodology (supporting); supervision (equal); writing – review and editing (equal). **Ahmed Ludvigsen Al‐Mashhadi:** Formal analysis (equal); methodology (equal); supervision (equal); writing – review and editing (equal). **Robert Schou Pedersen:** Formal analysis (equal); methodology (equal); supervision (supporting); writing – review and editing (equal). **Christian Bjørn Poulsen:** Formal analysis (equal); methodology (equal); supervision (equal); writing – review and editing (equal). **Anne Ortved Gang:** Formal analysis (equal); methodology (equal); supervision (equal); writing – review and editing (equal). **Peter Brown:** Formal analysis (equal); methodology (equal); supervision (equal); writing – review and editing (equal). **Tarec Christoffer El‐Galaly:** Formal analysis (equal); methodology (equal); supervision (equal); writing – review and editing (equal). **Thomas Stauffer Larsen:** Conceptualization (equal); formal analysis (equal); methodology (equal); resources (equal); supervision (equal); visualization (equal); writing – review and editing (equal).

## CONFLICT OF INTEREST STATEMENT

TSL (Consultancy/Advisory Board for BMS, Novartis, Gilead, Roche; Research Grant: Genentech; Travel Expenses: Roche); PB (Advisory board for Roche, Gilead, Swedish orphan, and SERB). Other authors have no conflict of interest to report.

## ETHICAL APPROVAL STATEMENT

Exemption from informed consent was granted by formal Danish Law and described in the guideline ‘Report on the use of clinical quality data for research’ that was written by the working group set up by the Advisory Board for Clinical Quality Development Program (RKKP). Details are described in ‘The Declaration on passing personal data covered by §10, a subsection of the Data Protection Act. 1 and 2’ https://www.retsinformation.dk/eli/lta/2019/1509. RKKP approved the study under the number 21/27006.

## Supporting information


Tables S1–S6.

## Data Availability

The datasets analyzed during the current study can be made available to the corresponding author upon reasonable request.
