# Supplementary material for: Revisiting beta‐2 microglobulin as a prognostic marker in diffuse large B‐cell lymphoma
Source: Cancer Med. 2024 Jun 18;13(12):e7239. doi: 10.1002/cam4.7239 (PMC11184650; doi:10.1002/cam4.7239)
Supplement: Supplementary file 1 — Tables S1–S6. [file CAM4-13-e7239-s001.docx]

**Suppl. Table** 1. Variables included in models analyzed in the current study, along with risk stratification and overall survival as provided in original publications, including newly developed β2M-NCCN-IPI.

| Model  (N of patients in original publications) | Model variables (points) | Risk groups (points) | % of patients | OS-years | OS (%) |
| --- | --- | --- | --- | --- | --- |
| IPI ^3^  (N=2,031) | Age >60 (1)  ECOG PS >1 (1)  Stage III/IV (1)  EN sites >1 (1)  LDH >ULN (1) | L (0-1)  LI (2)  HI (3)  H (4-5) | 35.0  27.0  22.0  16.0 | 5- | 73.0  51.0  43.0  26.0 |
| aaIPI ^3^  >60 years  (N=761) | Stage III/IV (1)  EN sites >1 (1)  LDH >ULN (1) | L (0)  LI (1)  HI (2)  H (3) | 18.0  31.0  35.0  16.0 | 5- | 56.0  44.0  37.0  21.0 |
| R-IPI ^24^  (N=365) | Age >60 (1)  ECOG PS >1 (1)  Stage III/IV (1)  EN sites >1 (1)  LDH >ULN (1) | very good (0)  good (1-2)  poor (3-5) | 10.0  45.0  45.0 | 4- | 94.0  79.0  55.0 |
| NCCN-IPI ^5^  (N=1,650) | Age ≤40 (0), 41-60 (1), 61-75 (2), >75 (3)  ECOG PS >1 (1)  Stage III/IV (1)  EN sites ≥1 (1)  LDH >1-3xULN (1), >3xULN (2) | L (0-1)  LI (2-3)  HI (3-4)  H (6-8) | 19.0  42.0  31.0  8.0 | 5- | 96.0  82.0  64.0  33.0 |
| β2-IPI ^16^  (N=71) | Age >60 (1)  ECOG PS >1 (1)  Stage III/IV (1)  EN sites >1 (1)  LDH >ULN (1)  β2M >ULN (1) | L (0-1)  LI (2)  HI (3)  H (4-6) | 39.4  8.4  8.4  43.7 | 3- | ~75.0  ~62.0  ~33.5  ~8.5 |
| GELTAMO-IPI ^20^  (N=1,848) | Age <65 (0), 65-79 (1), ≥80 (2)  ECOG PS 0-1 (0), 2 (1), 3-4 (2)  Stage III/IV (1)  LDH >ULN (1)  β2M >ULN (1) | L (0)  LI (1-3) HI (4)  H (5-7) | 11.0  58.0  17.0  14.0 | 5- | 93.0  79.0  66.0  39.0 |
| Modified Prognostic Model ^18^  (N=621) | Age >60 years (1)  ECOG PS >1 (1)  Stage III/IV (1)  LDH >ULN (1)  β2M >2.5 mg/L (1) | L (0)  LI (1) HI (2-3)  H (4-5) | 23.5  24.6  37.5  14.3 | 5- | 95.2  86.4  69.2  47.8 |
| New Prognostic  Index ^17^  (N=274) | Age >60 years (1)  ECOG PS >1 (1)  Stage III/IV (1)  β2M ≥3.2mg/L (1) | L (0)  LI (1-2) HI (3)  H (4) | 9.5  59.1  18.2  13.1 | 3- | 100.0  87.0  57.2  23.4 |
| β2M-NCCN-IPI  (N=3,232) | Age ≤40 (0), 41-60 (1), 61-75 (2), >75 (3)  ECOG PS >1 (1)  Stage III/IV (1)  EN sites ≥1 (1)  LDH >1-3xULN (1), >3xULN (2)  β2M >ULN (1) | L (0-1)  LI (2-4)  HI (5-6)  H (7-9) | 8.0  48.1  32.7  11.2 | 5- | 96.4  82.4  56.2  33.1 |

^aaIPI – age-adjusted IPI; ECOG PS – Eastern Oncology Cooperative Group performance status; EN – Extranodal; GELTAMO-IPI – Grupo Español de Linfomas y Trasplantes de Médula Ósea IPI; H – high; I – intermediate; IPI – International Prognostic Index; L – low; LDH – lactate dehydrogenase; LI – low-intermediate; NCCN-IPI – National Comprehensive Cancer Network-IPI; N – number; OS – overall survival; R-IPI – Revised International Prognostic Index; ULN – upper limit of normal; β2M – beta-2 microglobulin^

**Suppl. Table 2**. Logistic regression analysis for the association between clinical factors and β2M (≤ULN vs. >ULN) as dependent variable.

|  | Univariate analysis |  | Multivariate analysis with NCCN-IPI variables | |
| --- | --- | --- | --- | --- |
| Variables | OR, 95 % CI | p-value | OR, 95% CI | p-value |
| Gender | 0.954 (0.830; 1.097) | **0.507** |  |  |
| Age | 1.056 (1.050; 1.063) | <0.001 | 1.054 (1.046; 1.062) | <0.001 |
| Stage I/II vs. III/IV | 4.228 (3.614; 4.945) | <0.001 | 2.699 (2.180; 3.342) | <0.001 |
| ECOG PS 0-1 vs. ≥2 | 4.024 (3.181; 5.090) | <0.001 | 2.112 (1.545; 2.888) | <0.001 |
| EN sites (NCCN-IPI) <1 vs. ≥1 | 2.426 (2.072; 2.840) | <0.001 | 1.497 (1.210; 1.853) | <0.001 |
| LDH ≤ ULN  >1-3xULN  >3x ULN | Reference  2.463 (2.124; 2.855)  8.639 (6.035; 12.365) | <0.001  <0.001 | Reference  2.410 (1.971; 2.947)  7.010 (4.470; 10.992) | <0.001  <0.001 |
| Bulky disease (> 10cm) | 1.868 (1.598; 2.183) | <0.001 | 1.276 (1.033; 1.575) | 0.023 |
| Creatinine μmol/L ꝉ | 1.030 (1.026; 1.034) | <0.001 | 1.034 (1.029; 1.039) | <0.001 |
| Other malignancy ‡ | 1.752 (1.392; 2.207) | <0.001 | 1.538 (1.158; 2.043) | 0.003 |

^ECOG PS – Eastern Oncology Cooperative Group performance status; EN – Extranodal; LDH – lactate dehydrogenase; NCCN-IPI – National Comprehensive Cancer Network International Prognostic Index; OR – odds ratio; ULN – upper limit of normal; β2M – beta-2 microglobulin^

^ꝉ risk estimations are produced for each unit of measurement^

^‡ excluding skin cancer other than melanoma^

**Suppl. Table 3**. Agreement between the risk groups of IPI, NCCN-IPI, and other clinical models with four risk groups evaluated using weighted Cohen κ.

| NCCN-IPI (N=3,232) | | | | |
| --- | --- | --- | --- | --- |
| Prognostic  models | N misclassified patients (%) | N misclassified patients  within NCCN-IPI risk groups (%) | N equally classified patients within NCCN-IPI (%) | weighted κ |
| IPI ^3^ | 1,298 (40.2) | 3 (1.1)  692 (55.6)  555 (42.4)  48 (11.9) | 271 (98.9)  553 (44.4)  755 (57.6)  355 (88.1) | 0.630 (0.614; 0.646) |
| aaIPI ^3^ | 1,541 (47.7) | 45 (16.4)  734 (59.0)  592 (45.2)  170 (42.2) | 229 (83.6)  511 (41.0)  718 (54.8)  233 (57.8) | 0.510 (0.491; 0.529) |
| β2-IPI ^16^ | 1,857 (57.5) | 9 (3.3)  720 (57.8)  877 (66.9)  251 (6.2) | 265 (96.7)  525 (42.2)  433 (33.1)  378 (93.8) | 0.554 (0.538; 0.570) |
| GELTAMO-IPI ^20^ | 1,088 (33.7) | 48 (17.5)  65 (5.2)  846 (64.6)  129 (32.0) | 226 (82.5)  1,180 (94.8)  464 (35.4)  274 (68.0) | 0.612 (0.592; 0.633) |
| Modified Prognostic Model ^18^ | 1,380 (42.7) | 49 (17.9)  745 (59.8)  557 (42.5)  29 (7.2) | 225 (82.1)  500 (40.2)  753 (57.5)  374 (92.8) | 0.557 (0.537; 0.577) |
| New Prognostic Index ^17^ | 1,191 (36.8) | 15 (5.5)  160 (12.8)  801(61.1)  215 (53.3) | 259 (94.5)  1,085 (88.0)  509 (38.9)  188 (46.7) | 0.566 (0.544; 0.587) |
| β2M-NCCN-IPI | 353 (10.9) | 0 (0%)  311 (20.0)  42 (4.0)  0 (100) | 258 (100.0)  1,245 (80.0)  1,015 (96.0)  361 (100.0) | 0.873 (0.860; 0.887) |

^aaIPI – age-adjusted IPI; GELTAMO-IPI – Grupo Español de Linfomas y Trasplantes de Médula Ósea IPI; IPI – International Prognostic Index; N – number; NCCN-IPI – National Comprehensive Cancer Network-IPI; β2M – beta-2 microglobulin^

^Cohen κ: <0.00 poor; 0-0.2 slight; 0.21-0.40 fair; 0.41-0.60 moderate; 0.61-0.80 substantial; 0.81-1.00 almost perfect agreement^

**Suppl. Table 4**. Univariate and multivariate analysis of IPI/NCCN-IPI and laboratory variables concerning progression-free survival.

|  | Univariate analysis |  | Multivariate analysis with IPI variables | | Multivariate analysis with NCCN-IPI variables | |
| --- | --- | --- | --- | --- | --- | --- |
| Variables | HR, 95 % CI | p-value | HR, 95% CI | p-value | HR, 95% CI | p-value |
| Age ≤60 vs. >60 years | 4.041 (3.420; 4.776) | <0.001 | 3.346 (2.822; 3.967) | <0.001 |  |  |
| Age ≤40  41-60  61-75  >75 | Reference  4.503 (2.298; 8.821)  11.703 (6.058; 22.609)  25.311 (13.078; 48.984) | <0.001  <0.001  <0.001 |  |  | Reference  3.714 (1.894; 7.281)  8.960 (4.631; 17.338)  16.869 (8.684; 32.770) | <0.001  <0.001  <0.001 |
| Stage I/II vs. III/IV | 1.907 (1.680; 2.165) | <0.001 | 1.204 (1.044; 1.390) | 0.011 | 1.230 (1.065; 1.420) | 0.005 |
| ECOG PS 0-1 vs. ≥2 | 3.099 (2.733; 3.513) | <0.001 | 2.181 (1.912; 2.488) | <0.001 | 1.975 (1.728; 2.256) | <0.001 |
| No EN sites 1 vs. >1 | 1.392 (1.241; 1.561) | <0.001 | 1.021 (0.903; 1.154) | **0.745** |  |  |
| EN sites (NCCN-IPI) <1 vs. ≥1 | 1.510 (1.347; 1.693) | <0.001 |  |  | 1.100 (0.973; 1.283) | **0.128** |
| LDH IPI normal vs. >ULN | 1.533 (1.372; 1.713) | <0.001 | 1.178 (1.046; 1.327) | 0.007 |  |  |
| LDH ≤ ULN  >1-3xULN  >3x ULN | Reference  1.420 (1.263; 1.595)  2.282 (1.901; 2.740) | <0.001  <0.001 |  |  | Reference  1.224 (1.105; 1.416)  1.434 (1.181; 1.743) | <0.001  <0.001 |
| β2M >ULN yes vs. no | 3.111 (2.754; 3.514) | <0.001 | 2.106 (1.848; 2.399) | <0.001 | 1.822 (1.596; 2,081) | <0.001 |
| β2M <3.2mg/L yes vs. no | 3.001 (2.684; 3.354) | <0.001 |  |  |  |  |
| β2M <2.5mg/L yes vs. no | 2.984 (2.646; 3.365) | <0.001 |  |  |  |  |

^CI – Confidence interval; ECOG PS – Eastern Oncology Cooperative Group performance status; EN – Extranodal; GELTAMO-IPI – Grupo Español de Linfomas y Trasplantes de Médula Ósea IPI; HR – Hazard ratio; LDH – lactate dehydrogenase; NCCN-IPI – National Comprehensive Cancer Network-IPI; ULN – upper limit of normal; β2M – beta-2 microglobulin^

**Suppl. Table 5**. Summary of hazard ratios, overall performance, fit/quality, and discrimination measures concerning progression-free survival.

|  | HR (95% CI) | IBS | AIC | BIC | CPE | AUC | c-index | Difference in c-index with NCCN-IPI as referent model |
| --- | --- | --- | --- | --- | --- | --- | --- | --- |
| IPI ^3^ | Reference  1.671 (1.400; 1.996)  2.432 (2.057; 2.876)  4.629 (3.918; 5.469) | 0.203 | 19,032 | 19,047 | 0.638 | 0.638 | 0.670 (0.653; 0.687) | -0.019 (-0.028; -0.010) |
| aaIPI ^3^ | Reference  1.447 (1.210; 1.730)  1.893 (1.606; 2.231)  2.565 (2.143; 3.070) | 0.217 | 19,276 | 19,291 | 0.585 | 0.588 | 0.613 (0.599; 0.626) | -0.076 (-0.089; -0.063) |
| R-IPI ^24^ | Reference  6.183 (3.757; 10.180)  13.125 (7.996; 21.540) | 0.203 | 19,047 | 19,058 | 0.630 | 0.630 | 0.641 (0.625; 0.657) | -0.047 (-0.057; -0.038) |
| NCCN-IPI ^5^ | Reference  5.353 (3.334; 8.596)  11.772 (7.364; 18.817)  25.158 (15.599; 40.576) | 0.193 | 18,837 | 18,852 | 0.669 | 0.661 | 0.689 (0.672; 0.705) | **Reference** |
| β2-IPI ^16^ | 1.963 (1.568; 2.458)  2.656 (2.152; 3.279)  5.472 (4.519; 6.626) | 0.198 | 18,938 | 18,954 | 0.658 | 0.653 | 0.683 (0.667; 0.698) | **-0.006 (-0.015; 0.003)** |
| GELTAMO-IPI ^20^ | Reference  4.779 (3.228; 7.075)  10.945 (7.327; 16.352)  23.606 (15.787; 35.297) | 0.187 | 18,746 | 18,762 | 0.659 | 0.663 | 0.689 (0.675; 0.703) | **-0.000 (-0.013; 0.013)** |
| Modified Prognostic Model ^18^ | Reference  3.393 (2.043; 5.634)  7.292 (4.500; 11.814)  16.539 (10.198; 26.823) | 0.194 | 18,885 | 18,900 | 0.664 | 0.655 | 0.682 (0.669; 0.695) | **-0.007 (-0.016; 0.003)** |
| New Prognostic Index ^17^ | Reference  4.964 (3.460; 7.120)  13.136 (9.110; 18.940)  22.662 (15.490; 33.16) | 0.189 | 18,767 | 18,782 | 0.667 | 0.662 | 0.687 (0.671; 0.706) | -**0.001 (-0.015; 0.012)** |
| β2M-NCCN-IPI | 5.815 (3.532; 9.575)  15.376 (9.347; 25.292)  28.996 (17.471; 48.122) | 0.189 | 18,756 | 18,772 | 0.676 | 0.673 | 0.700 (0.677; 0.722) | 0.011 (0.005; 0.017) |

^aaIPI – age-adjusted IPI; AIC – Akaike Information Criterion; AUC – Area under the curve; BIC – Bayesian Information Criterion; c-index – concordance index; CI – confidence interval; CPE – Concordance probability estimate; DLBCL – Diffuse large B-cell lymphoma; GELTAMO-IPI – Grupo Español de Linfomas y Trasplantes de Médula Ósea IPI; HR – hazard ratio; IBS – integrated Brier score; IPI – International Prognostic Index; NCCN-IPI – National Comprehensive Cancer Network-IPI; N – number; R-IPI – Revised International Prognostic Index; β2M – beta-2 microglobulin^

**Suppl. Table 6**. Summary of hazard ratios, overall performance, fit/quality, and discrimination measures concerning overall survival of β2M-NCCN-IPI, NCCN, and IPI in training and validation cohorts.

|  | β2M-NCCN-IPI  Training cohort  (N=2,155)  N (%) | NCCN-IPI  Training cohort  (N=2,155)  N (%) | IPI  Training cohort  (N=2,155)  N (%) | β2M-NCCN-IPI  Validation cohort  (N=1,077)  N (%) | NCCN-IPI  Validation cohort  (N=1077)  N (%) | IPI  Validation cohort  (N=1077)  N (%) |
| --- | --- | --- | --- | --- | --- | --- |
| L  LI  HI  H | 161 (7.5)  1,027 (47.7)  723 (33.5)  244 (11.3) | 174 (8.1)  803 (37.2)  909 (42.2)  269 (12.5) | 584 (27.1)  538 (25.0)  586 (27.2)  447 (20.7) | 97 (9.0)  529 (49.1)  334 (31.0)  117 (110.9) | 100 (9.3)  442 (41.0)  401 (37.2)  134 (12.4) | 318 (29.5)  275 (25.5)  287 (26.6)  197 (18.3) |
| 5-year OS (%)  L  LI  HI  H | 95.8  82.2  55.2  32.3 | 96.1  82.9  60.9  33.9 | 86.7  74.5  65.3  42.4 | 97.4  83.0  58.3  34.9 | 96.3  83.5  63.4  37.1 | 85.9  78.0  66.7  46.2 |
| HR (95% CI) | Reference  6.421 (3.308; 12.460)  16.850 (8.694; 32.660)  32.991 (16.855; 64.570) | Reference  6.009 (3.190; 11.320)  13.303 (7.104; 24.910)  29.885 (15.797; 56.540) | Reference  1.842 (1.479; 2.294)  2.473 (2.005; 3.050)  4.967 (4.035; 6.115) | Reference  4.785 (2.241; 10.210)  13.013 (6.106; 27.730)  24.751 (11.404; 3.720) | Reference  4.340 (2.123; 8.872)  9.411 (4.635; 19.112)  19.957 (9.662; 41.220) | Reference  1.330 (0.9816; 1.801)  2.240 (1.6940; 2.961)  4.082 (3.0787; 5.414) |
| IBS | 0.185 | 0.187 | 0.195 | 0.184 | 0.189 | 0.199 |
| AIC | 12,032 | 12,073 | 12,197 | 5,104 | 5,136 | 5,204 |
| BIC | 12,037 | 12,077 | 12,202 | 5,108 | 5,140 | 5,208 |
| CPE | 0.661 | 0.663 | 0.644 | 0.664 | 0.660 | 0.635 |
| AUC | 0.689 | 0.677 | 0.659 | 0.699 | 0.686 | 0.660 |
| c-index | 0.707 (0.690; 0.723) | 0.696 (0.676; 0.716) | 0.678 (0.656; 0.700) | 0.712 (0.675; 0.748) | 0.701 (0.671; 0.731) | 0.674 (0.657; 0.692) |
| Difference in c-index | Reference | **0.011 (0.005; 0.016)** | **0.028** **(0.011; 0.046)** | Reference | **0.011** **(0.002; 0.020)** | **0.037 (0.021; 0.054)** |

^AIC – Akaike Information Criterion; AUC – Area under the curve; BIC – Bayesian Information Criterion; c-index – concordance index; CI – confidence interval; CPE – Concordance probability estimate; H – high; HR – hazard ratio; IBS – integrated Brier score; IPI – International Prognostic Index; L – low; LI – low-intermediate; NCCN-IPI – National Comprehensive Cancer Network-IPI; OS – overall survival; N – number; β2M – beta-2 microglobulin^
